# Factors associated with medication interruption among outpatients with severe mental illness exposed to COVID-19

**DOI:** 10.3389/fpubh.2023.1086863

**Published:** 2023-03-28

**Authors:** Jian Jiao, Yuanyi Ji, Hua Ren, Yanni Hao, Xiaoling Shen, Zaiquan Dong

**Affiliations:** ^1^Sleep Medicine Center, Mental Health Center, Translational Neuroscience Center, West China Hospital, Sichuan University, Chengdu, China; ^2^West China School of Public Health, West China Fourth Hospital of Sichuan University, Chengdu, Sichuan, China; ^3^The Fourth People’s Hospital of Chengdu, Chengdu, Sichuan, China; ^4^Mental Health Center, West China Hospital of Sichuan University, Chengdu, Sichuan, China

**Keywords:** COVID-19, severe mental illness, schizophrenia, medication compliance, medication interruption

## Abstract

Many patients with severe mental illness (SMI) relapsed and deteriorated during the COVID-19 pandemic, as they experienced medication interruption. This study aimed to investigate factors affecting medication interruption in patients with SMI during the COVID-19 pandemic. A total of 2,077 patients with SMI participated in an online survey on medication interruption during the COVID-19 outbreak. The questionnaire comprised six parts: basic demographic information, COVID-19 exposure, state of disease, medication compliance before COVID-19, medication interruption during COVID-19, and the specific impact and needs. A total of 2,017 valid questionnaires were collected. Nearly 50% of patients with SMI have been affected to varying degrees of life expectancy and treatment. Among them, 74 patients stopped taking medicines for more than 14 days without a prescription. Logistic regression analysis showed that cohabitant exposure [OR = 26.629; 95% CI (3.293–215.323), *p* = 0.002], medication partial compliance and non-compliance pre-COVID-19 [OR = 11.109; 95% CI (6.093–20.251), *p* < 0.001; OR = 20.115; 95% CI (10.490–38.571), *p* < 0.001], and disease status [OR = 0.326; 95% CI (0.188–0.564), *p* < 0.001] were related to medication interruption. More than 50% of the patients wanted help in taking medications, follow-up, and receiving more financial support and protective materials. We found that the daily lives of patients with SMI were much more susceptible to impact during the pandemic. Patients with a history of partial or non-medication compliance before COVID-19 and an unstable disease state are more easily affected by pandemics and epidemics and need extra attention should similar large-scale outbreaks occur in the future.

## 1. Introduction

Almost 3 years have passed since the first confirmed case of coronavirus disease 2019 (COVID-19) was reported in Wuhan, China. Scientists now have a deeper understanding of COVID-19, including the natural origin of its causative agent (severe acute respiratory syndrome coronavirus 2: SARS-CoV-2) and mode of transmission (human-to-human transmission, etc.), and have developed several vaccines (1–4). As a result of concerted global efforts, the COVID-19 pandemic has been controlled in many parts of the world. This outstanding achievement is largely due to the strict, large-scale prevention and control mechanisms adopted by governments, such as the immediate implementation of lockdown measures, enforced home isolation and quarantine measures, wearing masks, and social (physical) distancing (5, 6).

However, these procedures have had a negative impact on psychological wellbeing, causing profound changes in health behaviors, including decreased daily physical activity and increased rates of anxiety, depression, irritability, and insomnia (7). Further findings suggest that people with pre-existing mental health disorders are more likely to develop adverse mental health effects, with their symptoms increasing during the pandemic (8). In addition, basic healthcare services that are important for maintaining and stabilizing clinical symptoms and ensuring long-term prognosis for chronic diseases such as severe mental illness (SMI), including routine physical examinations and regular follow-ups, were disrupted during the pandemic due to an unbalanced distribution of medical resources (9). Thus, patients with chronic diseases such as SMI may be the most vulnerable to the COVID-19 pandemic (10). It is generally accepted that medication maintenance is one of the most important ways to prevent relapse and rehospitalization for chronic diseases. It has been estimated that non-compliance leads to an approximately 40% chance of relapse in schizophrenia (11). Even short periods (2 weeks or less) of medication interruption can nearly double the risk of psychotic exacerbation and hospitalization (11, 12). In such cases, good medication compliance is particularly important for maintaining the patient’s condition and preventing relapse. For example, studies have found that although the frequency of seizures increased slightly during the pandemic, patients with epilepsy were more motivated and informed about drug compliance and had no impact on stigma (13).

Apart from consistent medication compliance, a 12-month follow-up study on substance use disorder (SUD) and major depressive disorder (MDD) found that general health for asymptomatic SUD and MDD, and physical functioning for SUD were two predictors of relapses (14). Furthermore, social support, another important factor in rehabilitation and prognosis, has been explored in several studies. However, its role may vary across different psychiatric diagnoses. A 1-year follow-up study found that social support was the only factor that predicted the presence of relapses in SUD with schizophrenia but not in SMI patients with major depressive disorder (15).

In China, SMI is the general term for a group of disorders, such as schizophrenia, schizoaffective disorder, paranoid psychosis, bipolar disorder, mental disorders caused by epilepsy, and intellectual disability (16, 17). In clinical practice, we have encountered patients with SMI who experienced medication interruption during COVID-19, which led to their relapse and varying degrees of deterioration in their illnesses. Patients with SMI often have obvious mental symptoms at the onset of the disease, such as hallucinations, delusions, serious thought disorders, and behavioral disorders. They lose self-awareness through the disease, and consequently, control over their behavior, endangering personal and public safety (18). Furthermore, caregivers, peers, and healthcare workers bear an additional burden, increasing the risk of infection in this special situation (9). However, there has been no investigation of the factors associated with medical interruption in patients with SMI during the COVID-19 pandemic.

Therefore, this study aimed to identify such patients’ medical interruption status and influencing factors during the COVID-19 pandemic. Furthermore, the study explores the unique needs of such patients in order to provide them with more targeted help. We hope that our work provides a theoretical basis for ensuring the stability of patients’ clinical symptoms in cases where similar outbreaks occur in the future.

## 2. Methods and analysis

### 2.1. Participants

Patients with SMI were recruited from four districts in Chengdu, Sichuan Province, China, where the pandemic occurred. All participants and legal guardians were aware of the contents of the informed consent form and willing to cooperate with the investigation. Verbal informed consent was obtained from all participants prior to enrolment. Combined with other studies on patients with mild cognitive impairment or involuntary mental illness, they require careful evaluation of decision-making ability before treatment and research (19–21). However, due to the strict, large-scale prevention and control mechanisms adopted by the Chinese government, the assessment of decision-making ability and on-site data collection of patients with severe mental illness have been seriously affected. Furthermore, patients with severe mental illness are vulnerable groups; most cannot agree to treatment and research, and they are determined to have limited to no capacity for civil conduct according to the Civil Code of the People’s Republic of China.^1^ Therefore, we sent the assessment questionnaires *via* the internet, to patients’ caregivers and legal guardians to assess their conditions. This was mainly to prevent the spread of COVID-19; caregivers and legal guardians were contacted because they have been involved in patients’ everyday care and are most familiar with their medicines and treatment. This study was approved by the ethics committee on Biomedical Research of West China Hospital and conducted in accordance with the Declaration of Helsinki.

### 2.2. Inclusion criteria

All patients met the diagnostic criteria for SMI according to the International Statistical Classification of Diseases and Related Health Problems 10th Revision (ICD-10) and were under community management by an administrator. Patients that had full capacity for civil conduct, as assessed by the Judicial Expertise Center, completed their own informed consent with their legal guardians. Patients with SMI had to take prescribed medicines regularly before COVID-19. Administrators have medical records of all patients in their jurisdictions.

### 2.3. Exclusion criteria

Patients with SMI or their legal guardians who were unable or unwilling to provide consent and those with no telephone or who were unable to operate the phone to cooperate with the investigation were excluded. Patients without legal guardians were not included in this study. If the patient’s condition had reached clinical remission and they had completely stopped taking medicine under the guidance of a doctor before the pandemic, they were also excluded.

### 2.4. Procedure

With reference to the management of patients with SMI and the on-site survey, questionnaires were compiled by five senior psychiatrists with professional qualifications. The questionnaires were further revised through a pre-survey and used for the interviews. To facilitate data collection and ensure data integrity, we conducted a survey *via* the Wenjuanxing platform^2^, which sounded a warning when there were unanswered questions at the time of submission. The questionnaire included six parts (31 items).

1. *Basic demographic information:* age (below 30 years, between 30 and 50 years, and older than 50 years), sex (male or female), education level (lower than senior high school, senior high school, college, or undergraduate), living status (not alone, living alone), marital status (single, married, divorced, or widowed), monthly family income, reimbursement ratio of drug insurance (none, partial, complete), working status (working or unemployed), and family relationships (poor, good).
2. *COVID-19 exposure* (yes or no).
3. *The state of disease* (diagnosis, medical history, disease state, social function assessment, insight, drug knowledge, side effect assessment, etc.)
4. *Medication compliance pre-COVID-19* (Complete compliance, partial compliance, and non-compliance).
5. *Medication interruption*, specifically examining the impact of the pandemic (e.g., medication type, days of interruption, and follow-up).
6. *The specific impact and needs of Patients with SMI during COVID-19*: Inconvenience in taking medicine, inconvenience in patient’s follow-up to hospital, inconvenience in doctor’s return visit, and so on.

The study was conducted in Chengdu, China, between 3 September and 7 October 2020, during a relatively stable phase of the pandemic.

### 2.5. Definitions and standards

To ensure data consistency, we set the following evaluation criteria according to previous literature:

1. *Medication interruption:* Any treatment gap of at least 14 days without prescription during COVID-19, including antipsychotics, antidepressants, and mood stabilizers (22).
2. *COVID-19 exposure:* The patient had a history of travel in epidemic areas, there was a confirmed patient in the residential community, or the patient’s cohabitant had a history of exposure to COVID-19 or was diagnosed with COVID-19.
3. *Evaluation of sojourn history:* Living or traveling in communities where confirmed cases or asymptomatic infected persons have been reported in China or having a travel history of visiting countries or regions with epidemic diseases within 14 days before the onset of illness.
4. *Living community exposure:* People in their community who had been diagnosed with COVID-19 in the preceding 2 weeks.
5. *Cohabitant’s exposure:* The patient’s cohabitant had a history of contact with COVID-19 or was diagnosed with COVID-19.
6. *Retrospective evaluation of medication compliance pre-COVID-19:* Complete compliance meant that the patient completely obeyed medical advice and insisted on taking medication; partial compliance referred to situations in which patients did not take medication according to medical advice consistently and took less, missed, took more, or failed to take medication on time. Non-compliance refers to cases in which patients fail to take medicines according to medical advice and often refuse to obey or stop taking medicines.
7. *Evaluation of social function:* Self-care ability, housework, productive labor and work, learning ability, social interpersonal communication, and so on. According to the patient’s actual situation, this was divided into three categories: completely, partly, and not at all.
8. *Apropos work status:* Working status described people who had been able to work for most of the preceding year and had a fixed income; otherwise, they were regarded as unemployed.
9. *Disease state*: The general state of diseases during COVID-19 was divided into stable and unstable, according to whether the disease state fluctuated. Insight refers to the patient’s cognitive ability in their own psychological state, which is divided into no insight, partial insight, or full insight.
10. *Regarding drug knowledge evaluation:* According to the patient’s understanding of the importance of drug maintenance therapy, the effect of drug treatment, common side effects, and precautions for administration, drug knowledge evaluation was divided into three situations: good, general, and poor.
11. *Regarding the evaluation of drug side effects:* The patients’ main complaints were divided into two types: yes or no.
12. *Drug type evaluation:* This refers to the number of different drugs taken by patients simultaneously.
13. *Regarding the evaluation of outpatient follow-up to the hospital:* Regular follow-ups referred to follow-ups according to the medical arrangement or self-arrangement; intermittent follow-ups referred to patients arranging irregular follow-ups according to the changes in their illness, although not according to the time agreed by their doctors. No follow-up meant that there was neither a doctor’s appointment nor any self-arranged follow-up.
14. *Family support status:* Good support referred to family income that could completely meet patients’ medication expenses. This generally described family income that could partially meet their medication expenses, and which did not cause great hardship to the family. Poor support meant that the family income could not meet medication expenses, resulting in great hardship for the family.
15. *Family relationship:* A good relationship referred to a comfortable and happy relationship with the family, where the patient felt respected and supported by family members. A poor relationship meant that the patient’s family was not harmonious, and showed little concern, respect, or understanding toward the patient.

### 2.6. Quality control

To familiarize administrators with the use of the questionnaires and to unify the evaluation criteria based on the definitions and standards described earlier, we conducted centralized training for all administrators who participated in the survey. We asked administrators to check the questionnaire surveys individually and verify them with caregivers and legal guardians. The Wenjuanxing platform had a warning system that ensured that the participants completed all survey questions. Each person could answer the questionnaire only once to ensure the authenticity and reliability of the questionnaire data. Data were entered by one person and verified by a second person. Any discrepancies in the data were verified by a third reviewer.

### 2.7. Statistical analysis

All statistical analyses were performed using SPSS 22.0. The significance level was set at α = 0.05, and all the tests were two-tailed. Categorical data are presented as n (%). Chi-square tests were used to identify potential predictive and associated factors. Logistic regression analysis was performed based on the inclusion criteria and variables, and the associations between risk factors and outcomes were presented as odds ratios (ORs) and 95% confidence intervals (CIs).

## 3. Results

### 3.1. General demographic information

There were 2,077 patients with SMI in four districts of Chengdu, China. Three patients were excluded as they refused to participate in the study. Fifty-five older adults who lived alone without legal guardians were also excluded because they either did not have a mobile phone or could not operate one. Most patients and legal guardians were willing to participate in the survey, which may be based on long-term contact and good relationships between community managers and patients or guardians. Two patients with SMI who were in stable condition and did not take medicine according to their doctor’s advice were excluded.

In total, 2,017 valid questionnaires were collected (Table 1). The respondents included 1,156 (57.3%) women and 861 (42.7%) men; 16.6% of the participants were under 30 years of age, 48.9% were between 30 and 50 years old, and 34.5% were 50 years old or above. Most patients were diagnosed with schizophrenia (*n* = 1,502, 74.5%), whereas the rest had bipolar disorder (*n* = 181, 9.0%), paranoid psychosis (*n* = 28, 1.4%), intellectual disability (*n* = 107, 5.3%), mental disorders caused by epilepsy (*n* = 129, 6.4%), and schizoaffective disorder (*n* = 70, 3.4%).

**Table 1:** Bivariate association between medication interruption and related factors.

Variables		*N*(%)	No medication interruption	Medication interruption	*χ* ^2^	*p-*value
Sex (*n =* 2,017)	Female	1,156(57.3)	1,117(57.5)	39(52.7)		
	Male	861(42.7)	826(42.5)	35(47.3)	0.667	0.414
Age, years (*n =* 2,017)	<30	334(16.6)	321(16.5)	13(17.6)		
	30–50	987(48.9)	954(49.1)	33(44.6)		
	>50	696(34.5)	668(34.4)	28(37.8)	0.590	0.745
Education level (*n =* 2,017)	<Senior high school	1,460(72.4)	1,409(72.5)	51(68.9)		
	Senior high school	306(15.2)	291(15.0)	15(20.3)		
	College or undergraduate	251(12.4)	243(12.5)	8(10.8)	1.609	0.447
Marital status (*n =* 2,017)	Single	615(30.5)	591(30.4)	24(32.4)		
	Married	1,134(56.2)	1,091(56.2)	43(58.1)		
	Divorced or widowed	268(13.3)	261(13.4)	7(9.5)	0.991	0.609
Living status (*n =* 2,017)	Not alone	1902(94.3)	1837(94.5)	65(87.8)		
	Alone	115(5.7)	106(5.5)	9(12.2)	5.964	0.015
Monthly household income (*n =* 2,017)	<5,000	1,448(71.8)	1,388(71.4)	60(81.1)		
	5,000–10,000	393(19.5)	381(19.6)	13(17.6)		
	>10,000	175(8.7)	174(9.9)	1(1.4)	5.826	0.054
Family relationships (*n =* 2,017)	Poor	164(8.1)	149(7.7)	15(20.3)		
	Good	1853(91.9)	1794(92.3)	59(79.7)	15.155	0.000
Working (*n =* 2,017)	Working	521(25.8)	503(25.9)	18(24.3)		
	Unemployed	1,496(74.2)	1,440(74.1)	56(75.7)	0.091	0.763
Reimbursement ratio of drug insurance (*n =* 2,017)	None	210(10.4)	199(10.2)	11(14.9)		
	Partial	1,631(80.9)	1,572(80.9)	59(79.7)		
	Total	176(8.7)	172(8.9)	4(5.4)	2.446	0.294
Epidemic exposure (*n =* 2,017)	No	1969(97.6)	1898(97.7)	71(95.9)		
	Yes	48(2.4)	45(2.3)	3(4.1)	0.927	0.336
Sojourn history (*n =* 2,017)	No	2008(99.6)	1935(99.6)	73(98.6)		
	Yes	9(0.4)	8(0.4)	1(1.4)	1.417	0.234
Living community exposure (*n =* 2,017)	No	1981(98.2)	1908(98.2)	73(98.6)		
	Yes	36(1.8)	35(1.8)	1(1.4)	0.082	0.774
Cohabitant’s exposure (*n =* 2,017)	No	2012(99.8)	1940(99.8)	72(97.3)		
	Yes	5(0.2)	3(0.2)	2(2.7)	18.720	0.000
Diagnosis (*n =* 2,017)	Schizophrenia	1,502(74.5)	1,448(74.5)	54(73.0)		
	Bipolar disorder	181(9.0)	172(8.9)	9(12.2)		
	Paranoid psychosis	28(1.4)	26(1.3)	2(2.7)		
	Intellectual disability	107(5.3)	105(5.4)	2(2.7)		
	Mental disorders caused by epilepsy	129(6.4)	128(6.6)	1(1.4)		
	Schizoaffective disorder	70(3.4)	64(3.3)	6(8.1)	10.647	0.059
Medical history (Year) (*n =* 2,017)	<1	76(3.8)	69(3.6)	7(9.5)		
	1–5	527(26.1)	508(26.1)	19(25.7)		
	5–10	604(29.9)	588(30.3)	16(21.6)		
	>10	810(40.2)	778(40.0)	32(43.2)	8.569	0.036
Disease state (*n =* 2,017)	Unstable	246(12.2)	220(11.3)	26(35.1)		
	Stable	1771(87.8)	1723(88.7)	48(64.9)	37.745	0.000
Side-effects assessment	No	1762(84.8)	1,664(85.6)	47(63.5)		
(*n =* 2,017)	Yes	306(15.2)	279(14.4)	27(36.5)	27.120	0.000
Kind of drugs (*n =* 2,017)	1	588(29.2)	555(28.6)	33(44.6)		
	2–5	1,416(70.2)	1,375(70.8)	41(55.4)		
	>5	13(0.6)	13(0.7)	0(0.0)	9.175	0.010
Medication compliance pre-COVID-19 (*n =* 2,017)	Complete compliance	1837(91.1)	1804(92.8)	33(44.6)		
	Partial compliance	114(5.7)	93(4.8)	21(28.4)		
	Non-compliance	66(3.3)	46(2.4)	20(27.0)	220.896	0.000
Personal self-care ability (*n =* 2,017)	Completely	1,199(59.4)	1,159(59.7)	40(54.1)		
	Partly	773(38.3)	744(38.3)	29(39.2)		
	Not at all	45(2.2)	40(2.1)	5(6.8)	7.443	0.024
Housework (*n =* 2,017)	Completely	1,025(50.8)	993(51.1)	32(43.2)		
	Partly	917(45.4)	879(45.2)	37(50.0)		
	Not at all	76(3.8)	71(3.7)	5(6.8)	3.044	0.218
Productive labor and work (*n =* 2,017)	Completely	807(40.0)	787(40.5)	20(27.0)		
	Partly	1,049(52.0)	1,008(51.9)	41(55.4)		
	Not at all	161(8.0)	148(7.6)	13(17.6)	12.249	0.002
Learning ability (*n =* 2,017)	Completely	684(33.9)	666(34.3)	18(24.3)		
	Partly	1,182(58.6)	1,138(58.6)	44(59.5)		
	Not at all	151(7.5)	139(7.2)	12(16.2)	9.912	0.007
Social interpersonal communication (*n =* 2,017)	Completely	702(34.8)	682(35.1)	20(27.0)		
	Partly	1,160(57.5)	1,119(57.6)	41(55.4)		
	Not at all	155(7.7)	142(7.3)	13(17.6)	11.158	0.004
Insight level (*n =* 2,017)	Full insight	1,272(63.1)	1,237(63.7)	35(47.3)		
	Partial insight	701(34.8)	671(34.5)	30(40.5)		
	No insight	44(2.2)	35(1.8)	9(12.2)	38.846	0.000
Drug knowledge (*n =* 2,017)	Good	618(30.6)	605(31.1)	13(17.6)		
	Moderate	1,188(58.9)	1,144(58.9)	44(59.5)		
	Poor	211(10.5)	194(10.0)	17(23.0)	15.784	0.000
Family support status (*n =* 2,017)	Poor	104(5.2)	98(5.0)	6(8.1)		
	average	712(35.3)	678(34.9)	34(45.9)		
	Good	1,201(59.5)	1,167(60.1)	34(45.9)	6.150	0.046
Follow-up (*n =* 2,017)	Regular follow-up	1,089(54.0)	1,064(54.8)	25(33.8)		
	Intermittent follow-up	705(35.0)	680(35.0)	25(33.8)		
	No follow-up	223(11.1)	199(10.2)	24(32.4)	24.529	0.000

A chi-square test with Fisher’s exact probability method was used.

The education level of most patients (*n* = 1,460, 72.4%) was lower than that of senior high school students, and most were unemployed (*n* = 1,496, 74.2%). Half of the participants were married (*n* = 1,134, 56.2%), and most were not living alone (*n* = 1,902, 94.3%). Most participants had an average monthly household income of 4,999 Chinese Yuan (CNY) (*n* = 1,448, 71.8%) and a good familial relationship (*n* = 1,853, 91.9%). In addition, more than 810 (40.2%) patients were diagnosed more than 10 years ago. A total of 1,416 (70.2%) patients were taking two to five medications. A total of 48 (2.4%) were exposed to COVID-19. Of these, nine (0.4%) had a history of travel in epidemic areas (e.g., Wuhan), 36 (1.8%) lived in COVID-19-infected communities, and five (0.2%) had a history of contact with COVID-19 (Table 1).

### 3.2. COVID-19’s impact on daily life and treatment among patients with SMI

We found that the daily lives of 1,121 (55.1%) patients were affected by COVID-19 at different levels, with 127 (6.3%) patients being seriously affected (Table 2). Compared to the other five severe mental diseases, patients with mental illness caused by epilepsy (less than 50%) were least affected by the pandemic. However, there was no statistical difference in the overall mean of the six groups of data, 99% CI (0.236–0.259), *p* = 0.247, by the chi-square test performed using the Monte Carlo method. In terms of treatment, 936 patients (46.6%) were affected. Among them, 56 (2.8%) were seriously affected, as assessed by the subjective feelings of patients’ families. However, the number of patients affected by COVID-19 was less than 50%. There was no statistical difference in the overall mean of the six groups of data, 99% CI (0.568–0.594), *p* = 0.581.

**Table 2:** Impact of COVID-19 on severe mental illness patients.

Item		Schizophrenia *n* (%)	Bipolar disorder *n* (%)	Paranoid psychosis *n* (%)	Intellectual disability *n* (%)	MD caused by epilepsy *n* (%)	Schizoaffective disorder *n* (%)	*N* (%)	*p-*value
The impact of COVID-19 on daily life	No obvious impact	664 (44.2)	80 (44.2)	13 (46.4)	50 (46.7)	69 (53.5)	29 (41.4)	905 (44.9)	
	General impact	738 (49.1)	88 (48.6)	14 (50)	56 (52.3)	55 (42.6)	34 (48.6)	985 (48.8)	
	Seriously impact	100 (6.7)	13 (7.2)	1 (3.6)	1 (0.9)	5 (3.9)	7 (10)	127 (6.3)	0.261
The impact of COVID-19 on treatment	No obvious impact	787 (52.4)	104 (57.5)	16 (57.1)	58 (54.2)	76 (58.9)	37 (52.9)	1,078 (53.4)	
	General impact	669 (44.5)	72 (39.8)	12 (42.9)	49 (45.8)	49 (38.0)	32 (45.7)	883 (43.8)	
	Seriously impact	46 (3.1)	5 (2.8)	0 (0.0)	0 (0.0)	4 (3.1)	1 (1.4)	56 (2.8)	0.588

MD, mental disorders. The chi-squared test was performed using the Monte Carlo method.

### 3.3. Specific impact and needs of patients with SMI during COVID-19

To help patients with SMI more effectively, we summarized the specific impacts and needs that emerged during the COVID-19 pandemic; it affected more than 50% of patients, resulting in difficulties in obtaining medications and follow-ups. More than 50% of the patients wanted to receive more financial support, protective materials, help in taking medicine, and more follow-up visits by their doctor (Table 3).

**Table 3:** Specific impacts and needs related to COVID-19.

Item		*N*	%	Item		*N*	%
Specific impacts of COVID-19 on medical behavior	Inconvenience in taking medicine	1,290	60.7	Specific needs in COVID-19	Convenience in taking medicine	1,334	66.1
	Inconvenience in patient’s follow-up to hospital	727	36.0		Convenience in follow-up to hospital	580	28.8
	Inconvenience in doctor’s return visit	1,081	50.8		Doctor’s return visit	1,146	56.8
					Financial support	1,037	51.4
					Convenience in using mental health services	650	32.2
					Using sufficient protection against COVID-19	1,055	52.3

### 3.4. Risk factors related to medication interruption

Seventy-four (4%) patients stopped taking medicines for more than 14 days without a prescription. The results of the bivariate analysis (Table 1) showed that, among all factors, medication interruption was related to the following 16 factors with statistical significance (all *p*-values less than 0.05): living conditions (*χ*^2^ = 5.964), family relationships (*χ*^2^ = 15.155), cohabitant exposure (*χ*^2^ = 18.720), medical history (*χ*^2^ = 8.569), disease state (*χ*^2^ = 37.745), side effects evaluation (*χ*^2^ = 27.120), several different categories of medication (*χ*^2^ = 9.175), medication compliance pre-COVID-19 (*χ*^2^ = 220.896), personal self-care ability (*χ*^2^ = 7.443), productive labor and work (*χ*^2^ = 12.249), learning ability (*χ*^2^ = 9.912), social interpersonal communication (*χ*^2^ = 11.158), insight level (*χ*^2^ = 38.846), drug knowledge (*χ*^2^ = 15.784), family support (*χ*^2^ = 6.150), and follow-up (*χ*^2^ = 24.529). To further clarify the relationship between the above factors and medication interruption, we performed a logistic regression analysis (Table 4). Medication interruption was set as the dependent variable, while these 16 factors were set as independent variables. By consulting experts and existing literature, we also included factors such as sex, age, education level, and residence as independent variables. The results showed that cohabitant exposure [OR = 26.629; 95% CI (3.293–215.323), *p* = 0.002], medication partial compliance pre-COVID-19 [OR = 11.109; 95% CI (6.093–20.251), *p* < 0.001], medication non-compliance pre-COVID-19 [OR = 20.115; 95% CI (10.490–38.571), p < 0.001], and disease status [OR = 0.326; 95% CI (0.188–0.564), p < 0.001] were closely related to medication interruption.

**Table 4:** Logistic regression analysis of factors associated with drug withdrawal.

Item	*B*	Sb	Wald	*p*	OR	95%CI
Cohabitants exposure	3.282	1.066	9.472	0.002	26.629	(3.293–215.323)
Medication partial compliance pre-COVID-19	2.408	0.306	61.755	0.001	11.109	(6.093–20.251)
Medication non-compliance pre-COVID-19	3.001	0.332	81.655	0.001	20.115	(10.490–38.571)
Stable disease state	−1.112	0.280	16.031	0.001	0.326	(0.188–0.564)

OR, odds ratio.

## 4. Discussion

In recent years, SMI has attracted increasing attention; it has become an important public health issue and a prominent social problem. As with other chronic diseases, positive health outcomes such as non-relapse or non-rehospitalization are heavily reliant on patterns of compliance with medicine, a relaxed environment, and appropriate psychological intervention (9). With the outbreak of COVID-19, countries have launched prevention and control mechanisms that can limit the spread of the epidemic; however, these have several side effects (6, 23). Among these, relapse and deterioration of SMI due to medication interruption during COVID-19 have elicited widespread concern.

We found that the daily life of more than 50% of patients with SMI was affected at different levels. Our results were higher than those reported in other studies; one study of Portuguese adults found that periods of social isolation resulted in altered eating habits, such as eating more (45.2%) or in larger quantities (31.6%) (24). A nationwide survey in the United States found that 27% of parents reported that their mental health deteriorated, while 14% reported that their children’s behavioral health deteriorated (25). However, a study on healthy Chinese adults who had been isolated at home for an average of 77 days found that more than 50% of respondents indicated that they spent less time on daily physical activity and more time on sedentary behaviors than before the lockdown, and 23% had changed their diet (6). Although we did not investigate the days of patients’ social isolation, we suggest that this difference may be due to the different durations of social isolation. Maintaining a consistent daily routine is important for patients with SMI to maintain good sleep and emotional stability. However, their daily lives are much more susceptible to change than that of a normal person, which is another important factor leading to treatment interruption or discontinuation. Therefore, when encountering similar situations in the future, we should pay more attention to those patients with SMI who have been severely affected by the government’s isolation policy and attempt to ensure that their normal routine is not compromised.

In addition, the treatment of nearly 50% of patients with SMI was affected. Among them, the condition of 246 (12.2%) patients fluctuated during the COVID-19 outbreak and 74 patients (4%) had a history of medication interruption. The rate of treatment discontinuation or interruption in 1 year was found to be between 40 and 75%, presenting a major obstacle to the effective treatment of schizophrenia (12, 26). The medication interruption rate of community-managed patients with SMI was much lower, which showed that community management could significantly improve medication compliance of patients with schizophrenia. This reminds us that strengthening the community management of patients with SMI may be an effective way to reduce and prevent the interruption of treatment. In addition, clinical practice should factor in the effects of the pandemic when evaluating compliance with drug treatment and the causes of disease fluctuation.

Binary logistic regression analysis indicated that medication interruption was related to cohabitant exposure, partial medication compliance before COVID-19, medication non-compliance before COVID-19, and disease status. It is well known that cohabitants often supervise and manage the drug treatment and follow-up of patients with SMI. According to the requirements of the pandemic prevention and control management policy in China^3^, when a cohabitant has been diagnosed with COVID-19, all patients should be transferred to their designated medical institutions for treatment and isolated medical observation within 2 h of the discovery. Cases that recovered after discharge should continue to be isolated for 14 days. Cohabitants (SMI patients) with confirmed COVID-19 patient were also considered to be in close contact with the novel coronavirus and underwent intensive isolated medical observation for 14 days. During this period, patients are often unable to regularly receive the necessary drugs. Therefore, we suggest that routine health surveys be conducted for infected family members and cohabitants to prevent similar situations from occurring again during the pandemic. For patients with SMI with one or more of the following conditions, including residents’ exposure, partial medication compliance, non-compliance, and unstable disease state, more attention should be paid to prevent medication interruption in the future when similar situations occur.

The literature indicates that non-suicidal self-injury is a strong predictor of future suicide attempts and behaviors (27, 28). This conceptual framework may also hold for medication interruptions. It is well known that medication non-compliance or partial compliance is the most important modifiable risk factor for relapse and rehospitalization in patients with schizophrenia (11, 29). Our results further showed that partial medication compliance pre-COVID-19 and non-compliance pre-COVID-19 may be related to medication interruption during the pandemic. Furthermore, a stable disease state was the only confirmed protective factor against medication interruption during the COVID-19 outbreak, which means that the more stable the patient, the lower the risk of medication interruption. Therefore, extra attention should be given to patients with an unstable disease state and a history of partial medication compliance or non-compliance before the pandemic.

This study had some limitations. First, drug types/differences could account for the variation in medication adherence across patients with various SMI. However, we did not factor this into our model (30). Second, owing to the requirements of pandemic prevention and control, we were limited to seeking anonymous responses to questionnaires online, which was the safest data collection option. In addition, the nature of the measures was such that they were based on retrospective recall, with most items being a single categorical variable rather than a psychometrically validated instrument. Furthermore, the current level of the participants’ functioning given their diagnoses is unclear, especially considering that some were described as having intellectual disabilities. Thus, the reliability of the data was unclear. Further research is required to confirm these findings.

## 5. Conclusion

This study demonstrates that, compared to healthy people, the daily life of patients with SMI was much more susceptible to the impact of the pandemic. Exposure to cohabitants and poor drug treatment compliance were closely related to medication interruption. Therefore, more attention should be paid to patients with SMIs in similar situations. This study provides empirical support for ensuring the stability of clinical symptoms of patients with SMI that should occur during similar epidemics or pandemics in the future.

## Data availability statement

The raw data supporting the conclusions of this article will be made available by the authors, without undue reservation.

## Ethics statement

The studies involving human participants were reviewed and approved by Ethics approval was granted by the ethics committee of the West China Hospital of Sichuan University [reference number 2020 (200)]. Written informed consent from the participants’ legal guardian/next of kin was not required to participate in this study in accordance with the national legislation and the institutional requirements.

## Author contributions

ZD received the funding and designed the study. JJ analyzed the data together with YJ. JJ drafted the manuscript. All authors were involved in this study, and have read and approved the final version of the manuscript.

## Funding

This study was supported by the Key R&D projects of the Science and Technology Department of Sichuan Province (grant number 2019YFS0217) and the 1.3.5 Project for Disciplines of Excellence, West China Hospital of Sichuan University.

## Conflict of interest

The authors declare that the research was conducted in the absence of any commercial or financial relationships that could be construed as potential conflicts of interest.

## Publisher’s note

All claims expressed in this article are solely those of the authors and do not necessarily represent those of their affiliated organizations, or those of the publisher, the editors and the reviewers. Any product that may be evaluated in this article, or claim that may be made by its manufacturer, is not guaranteed or endorsed by the publisher.

## Footnotes

^1^ http://www.npc.gov.cn/npc/c30834/202006/75ba6483b8344591abd07917e1d25cc8.shtml

^2^ https://www.wjx.cn/

^3^ http://www.gov.cn/fuwu/zt/yqfwzq/yqfkblt.htm
